# Persistent Pulmonary Hypertension of the Newborn in Very Low Birth Weight Infants: Risk Factors and Clinical Outcomes from a Matched Case–Control Study

**DOI:** 10.3390/jcm14134759

**Published:** 2025-07-04

**Authors:** Anucha Thatrimontrichai, Pattima Pakhathirathien, Manapat Praditaukrit, Gunlawadee Maneenil, Supaporn Dissaneevate, Ploypailin Jantarawongpisal, Jenjira Saechan

**Affiliations:** Department of Pediatrics, Division of Neonatology, Faculty of Medicine, Prince of Songkla University, Songkhla 90110, Thailand; ppattima@medicine.psu.ac.th (P.P.); manapat.p@psu.ac.th (M.P.); mgunlawa@medicine.psu.ac.th (G.M.); dsupapor@medicine.psu.ac.th (S.D.); ploypailin.ja@psu.ac.th (P.J.); jenjira.s@psu.ac.th (J.S.)

**Keywords:** Apgar score, asphyxia neonatorum, cerebral intraventricular hemorrhage, neonatal intensive care unit, newborn, nitric oxide, persistent pulmonary hypertension of the newborn, premature infant, respiratory distress syndrome, very low birth weight

## Abstract

**Background/Objectives**: To identify the risk factors and clinical outcomes of persistent pulmonary hypertension of the newborn (PPHN) in very low birth weight (VLBW) infants in a resource-limited setting. **Methods**: We conducted a 1:4 matched case–control study in a Thai neonatal unit between 2014 and 2023. Neonates born at a gestational age (GA) < 32 weeks and with a birth weight (BW) < 1500 g were included. Neonates who died in the delivery room or had major congenital anomalies were excluded. Matching was based on GA, BW, year of birth, and endotracheal intubation at birth. Conditional logistic regression analysis was performed. **Results**: Over the 10-year study period, the incidence of PPHN among VLBW neonates was 4.6% (31/667). After matching, there were 31 cases and 124 controls. In univariable analysis, PPHN was significantly associated with lower 1 min and 5 min Apgar scores; however, no significant association remained in multivariable analysis. PPHN was significantly associated with composite adverse outcomes—including mortality and major morbidities (adjusted odds ratio [aOR] = 7.51, 95% confidence interval [CI]: 2.41–23.40), mortality alone (aOR = 2.88, 95% CI: 1.06–7.63), major morbidities (aOR = 2.99; 95% CI: 1.29–6.95), and severe neurological injury (aOR = 4.44, 95% CI: 1.56–12.59). Daily hospital costs were also higher in PPHN cases, with an average increase of 97.1 USD. **Conclusions**: In VLBW infants, PPHN was associated with a lower Apgar score and surfactant administration. PPHN was significantly linked to adverse outcomes, particularly mortality, major morbidities, and severe neurological injury.

## 1. Introduction

Globally, very preterm neonates—defined as those born between 22^0/7^ and 31^6/7^ weeks of gestation—or those with very low birth weight (VLBW; <1500 g) are at significant risk of mortality and serious morbidities. Composite outcomes include death and major complications such as moderate to severe bronchopulmonary dysplasia (BPD), treated retinopathy of prematurity (ROP), and severe neurological injury (SNI), with reported incidence rates ranging from 21.8 to 60.0% for mortality and 3.4–22.0% for morbidity [1]. Key risk factors associated with mortality in VLBW neonates include low gestational age (GA), low Apgar scores, small-for-GA (SGA) status, and persistent pulmonary hypertension of the newborn (PPHN) [1,2].

PPHN is a life-threatening respiratory condition associated with the failure of normal fetal-to-neonatal circulatory transition, often requiring off-label use of pulmonary vasodilators in both preterm and term neonates. In previous studies, reported incidence rates of pulmonary hypertension in preterm neonates vary widely—from 0% to 11.5%—largely due to inconsistent diagnostic definitions, heterogeneous study populations, and differing study designs [2,3,4]. Most prior research has focused on early pulmonary hypertension (EPH), typically diagnosed via echocardiography, rather than on PPHN, which necessitates both clinical and echocardiographic criteria. Furthermore, these studies predominantly originated from high-income countries [5].

A study estimated the incidence of EPH in preterm neonates at 23.5%, though substantial heterogeneity was noted (I^2^ = 88.7%) [5]. Identified risk factors for EPH included oligohydramnios and SGA status [5]. The proportion of neonates receiving surfactant therapy was higher in neonates with PPHN than in those without [2,6]. Documented clinical outcomes associated with EPH include in-hospital mortality, moderate to severe BPD, and late-onset pulmonary hypertension [5]. However, few studies have specifically investigated the risk factors and outcomes of PPHN in VLBW infants, particularly in resource-limited settings. This study was therefore conducted in a Thai neonatal intensive care unit (NICU) to identify the risk factors and clinical outcomes associated with PPHN in this vulnerable population.

## 2. Materials and Methods

### 2.1. Setting and Population

This observational study was conducted in the NICU of Songklanagarind Hospital, a teaching hospital affiliated with the Prince of Songkla University, Thailand. The hospital records 2500–3500 live births annually, with 400–550 neonates admitted to its level IV NICU each year. The study protocol was approved by the Human Research Ethics Committee of Prince of Songkla University (Approval No.: 56–391–01–1).

The study population comprised very preterm and VLBW neonates born at Songklanagarind Hospital between 1 January 2014 and 31 December 2023. Neonates who died in the delivery room or had major congenital anomalies—defined according to Vermont Oxford Network criteria [7]—were excluded.

### 2.2. Definitions

PPHN was diagnosed within the first 14 days of life, based on both clinical and echocardiographic criteria. Clinically, affected neonates presented with labile hypoxemia and pre-to-post ductal differential cyanosis, defined as an oxygen saturation difference greater than 5–10% or a partial pressure of oxygen (PaO_2_) difference greater than 10–20 mm Hg. Echocardiographic findings supporting the diagnosis included increased pulmonary pressures with demonstrable right-to-left shunting across the ductus arteriosus or foramen ovale [8], interventricular septal flattening [8,9,10,11,12,13], or a right ventricular systolic pressure-to-systemic systolic pressure ratio exceeding 0.5 [9,10,14,15]. All neonates who met the clinical criteria for PPHN were first screened by trained neonatal fellows using echocardiography and were subsequently diagnosed by a pediatric cardiologist.

SGA was defined as a birth weight (BW) below the 10th percentile for GA. Preterm premature rupture of membranes (PPROM) was defined as rupture of the fetal membranes occurring more than 18 h before delivery and before 37 weeks of gestation. Surfactant therapy was administered to preterm neonates diagnosed with moderate or severe respiratory distress syndrome (RDS) who required a fraction of inspired oxygen (FiO_2_) exceeding 0.3 while on continuous positive airway pressure (CPAP) or mechanical ventilation via endotracheal intubation.

Regarding composite outcomes of mortality and major morbidities (including moderate or severe BPD, treated ROP, and SNI) in VLBW infants, mortality was defined as in-hospital mortality before discharge, excluding deaths in the delivery room. Moderate or severe BPD was defined as oxygen supplementation for at least 28 days, with continued need for supplemental oxygen and/or positive pressure respiratory support at 36 weeks postmenstrual age or at discharge, whichever came first [16]. Ocular examinations and cranial ultrasonography were performed only in neonates with clinical indications for screening [17,18]. Infants treated with laser surgery or intravitreal anti-vascular endothelial growth factor (anti-VEGF) therapy were classified as treated ROP. SNI was defined as grades III–IV intraventricular hemorrhage (IVH) and/or periventricular leukomalacia occurring after PPHN diagnosis. Daily hospital costs were calculated as total hospital charges divided by the length of hospital stay (1 USD = 35 Thai Baht).

### 2.3. VLBW Care

Neither delayed umbilical cord clamping nor minimally invasive surfactant administration (i.e., “Less invasive surfactant administration or the “Intubation Surfactant-Extubation” technique) was routinely performed in preterm infants during the study period. In VLBW infants with moderate-to-severe RDS, surfactant replacement therapy was generally initiated within 2–4 h after birth. High-frequency oscillatory ventilation (HFOV) was routinely employed as the primary ventilation mode after intubation. Target oxygen saturation was maintained at 90–94%.

Indomethacin for IVH prophylaxis was administered to neonates with hemodynamic stability and either a GA of less than 26 weeks or a BW under 750 g. Intravenous aminophylline was routinely administered to VLBW infants within the first week of life and transitioned to oral caffeine once full enteral feeding was achieved [19]. Postnatal systemic corticosteroids, following the “Dexamethasone: A Randomized Trial” (DART) protocol, or inhaled corticosteroids (fluticasone propionate via metered-dose inhaler) were considered after the first week of life to support extubation and facilitate oxygen weaning during noninvasive ventilation, respectively.

Routine echocardiographic screening was not performed in VLBW infants during the study period. Management of pulmonary hypertension in preterm infants was mainly supportive and ventilatory. In cases of clinical deterioration or severe pulmonary hypertension unresponsive to standard care, pulmonary vasodilator therapy—including inhaled nitric oxide (iNO) and/or oral agents—was considered at the discretion of the attending neonatologist.

### 2.4. Statistical Analysis

The R program (version 4.4.1; R Foundation for Statistical Computing, Vienna, Austria) was used to analyze the data. Categorical variables and non-parametric continuous variables were compared using the χ^2^ test or Fisher’s exact test, and the Mann–Whitney U test, respectively.

To perform matched case–control analysis, we used the MatchIt package (version 4.7.2) for matching and conditional logistic regression in R for subsequent analysis. The MatchIt package provides multiple matching algorithms; we employed nearest neighbor and Mahalanobis distance matching to select controls. Four controls were matched to each case (4:1 ratio), based on GA, BW, year of birth, and the use of endotracheal intubation at birth.

Conditional logistic regression was performed to compare data between VLBW neonates with and without PPHN. Independent variables (*p* < 0.2) in univariable analysis, or those identified as significant from previous studies, were retained in the multivariable model. Adjusted odds ratios (aORs) and confidence intervals (CIs) were reported for variables independently associated with PPHN.

## 3. Results

During the 10-year study period (2014–2023), 707 very preterm and VLBW neonates were initially assessed. Forty neonates were excluded due to congenital heart anomalies (*n* = 12), gastrointestinal anomalies (*n* = 21), chromosomal abnormalities (*n* = 5), or other congenital conditions (*n* = 2; congenital diaphragmatic hernia and hydrops fetalis). Thus, a total of 667 VLBW neonates were included in the final analysis (Table 1). The incidence of PPHN among VLBW neonates was 4.6% (31/667). The median age at echocardiographic diagnosis of PPHN was 3 days (interquartile range: 0.75–3). iNO was administered to 11 neonates (36%) with PPHN. The mortality rates among VLBW neonates with and without PPHN were 45.2% (14/31) and 10.1% (64/636), respectively.

After 1:4 case–control matching, risk factors and clinical outcomes of VLBW neonates with and without PPHN are shown in Table 2 and Table 3, respectively. In univariable analysis (Table 2), both 1 min and 5 min Apgar scores differed significantly between groups.

According to additional univariable analyses (Table 3), VLBW neonates diagnosed with PPHN had significantly higher rates of adverse clinical outcomes—including composite outcomes of mortality and major morbidities, individual mortality, major morbidities, and SNI, as well as longer durations of ventilator use and higher daily hospital costs compared to those without PPHN. The estimated additional daily hospital cost for neonates with PPHN was approximately 97.1 USD per day.

Conditional logistic regression analysis revealed that the risk factors associated with PPHN included lower 1 min and 5 min Apgar scores, SGA status, large-for-gestational-age (LGA) status, and surfactant administration. However, none of these factors demonstrated statistically significant differences between VLBW infants with and without PPHN (Table 4).

Significant variables from the univariable analysis (1 min and 5 min Apgar scores), along with key variables identified in previous studies (SGA status and surfactant administration), were included in the multivariable model (Table 5). The clinical outcomes of VLBW neonates with PPHN were significantly associated with increased odds of the composite outcome of mortality and major morbidities, individual mortality, major morbidities, and SNI. However, moderate or severe BPD and treated ROP were not significantly associated with PPHN.

## 4. Discussion

The incidence of VLBW neonates diagnosed with PPHN in this study was 4.6%. However, the definition and timing of PPHN diagnosis remain inconsistent across the literature. The reported incidence depends on the diagnostic criteria used, with lower rates observed when both clinical and echocardiographic criteria are applied, compared to echocardiographic criteria alone. A recent meta-analysis reported an EPH incidence of 23.5% in preterm neonates [5]. When PPHN is defined using both clinical and echocardiographic criteria, the incidence in preterm infants was reported as 3.0% [12], 4.6% (this study), and 8.1% [6]. Notably, the incidence of PPHN is inversely proportional to GA, with reported rates of 3.0%, 4.6%, and 8.1% at GAs below 34, 32, and 28 weeks, respectively [6,12]. The timing of EPH diagnosis varies, ranging from 3 to 5 days to 7–14 days after birth [2,11,13,14,20]. Elevated pulmonary artery pressure during the first 3 days of life may reflect normal physiological adaptation. Echocardiographic screening in asymptomatic neonates is typically performed between days 3 and 5 of life. For symptomatic neonates, the median age at echocardiographic diagnosis of PPHN was 1 (1–1) day in a Canadian study [2] and 3 (0.75–3) days in our study. The diagnostic criteria, indications for evaluation, and follow-up strategies—including the use of iNO and pulmonary vasodilators—in VLBW neonates should be explored in future studies.

In this study, PPHN was associated with lower 1 min and 5 min Apgar scores. Perinatal asphyxia, as reflected in low Apgar scores, may interfere with the normal transition from fetal to neonatal circulation, leading to delayed reduction in pulmonary vascular resistance rather than intrinsic pulmonary vascular disease [5]. Previous studies consistently reported lower 1 min [2,6,8,12] and 5 min [8] Apgar scores in neonates with PPHN compared to those without. Therefore, VLBW infants with low Apgar scores should be closely monitored for clinical signs of PPHN and considered for routine echocardiographic screening within the first 3 days of life.

VLBW neonates with PPHN in this study were significantly more likely to experience adverse clinical outcomes, including mortality, major morbidities, and SNI, compared to those without PPHN. These associations were consistent with previous studies that reported increased risks of composite outcomes of mortality and major morbidities [2,12], mortality [2], and IVH grades III–IV [12,21] in preterm neonates with PPHN. The mortality rate among VLBW neonates without PPHN in this study (10.1%) aligns closely with previous reports (9%) [8,10,11,12,13,14]. The increased rates of SNI in PPHN cases may be related to cardiorespiratory failure, including prolonged ventilator support and iNO use. The median duration of mechanical ventilation in neonates with PPHN in this study was 9 days, shorter than the 16 days reported in previous studies [2,12]. Similarly, the proportion of VLBW neonates with PPHN treated with iNO in this study (36%) was lower than previously reported rates of 43–92% [8,12,22,23]. Given the risk of mortality and neurodevelopmental complications, VLBW infants diagnosed with PPHN should receive close follow-up by pediatricians and caregivers. Early intervention programs and long-term neurodevelopmental assessments are recommended for these high-risk neonates.

Nevertheless, the interpretation and generalization of this study’s findings should be approached with caution due to some limitations. First, there may have been selection bias, resulting from the exclusion of neonates with mild PPHN. No routine screening or early functional echocardiography was conducted in any VLBW neonates, and idiopathic PPHN was not confirmed through cardiac catheterization. Second, several potential confounders—such as oligohydramnios, pulmonary hypoplasia, duration of prolonged PPROM, cord blood gas, placental pathology or dysfunction, vascular anomalies, maternal medication exposure, whole-genome sequencing, clinical risk index for babies (CRIB) score, ventilator settings, oxygenation index and vasoactive-inotropic score—were not evaluated. Third, there are no set standards of care or specific guidelines for the management of PPHN in VLBW infants, either in our center or globally. This includes a lack of clear recommendations regarding indications for PPHN screening, the use of iNO and other pulmonary vasodilators, and standardized protocols for the long-term follow-up of neonates with PPHN or those exposed to off-label medications. Finally, this study did not include long-term neurodevelopmental outcomes in VLBW infants with PPHN, owing to the high incidence of SNI.

## 5. Conclusions

This study demonstrated that VLBW neonates diagnosed with PPHN in a NICU in Thailand experienced significantly higher mortality, morbidity, and medical costs during admission. However, the risk factors and outcomes of PPHN are multifactorial and may be influenced by variations in ventilatory strategies, insurance coverage, and medical resource availability. A standardized diagnostic approach—along with clearly defined screening protocols and management strategies for EPH, PPHN, and late-onset pulmonary hypertension in preterm infants—is essential for developing effective clinical guidelines. Such frameworks are crucial for improving care quality and reducing preventable mortality and morbidity in this high-risk population.

## Figures and Tables

**Table 1:** Risk factors associated with persistent pulmonary hypertension of the newborn (PPHN) in the overall cohort.

Risk Factor	Neonates with PPHN (*n* = 31)	Neonates Without PPHN (*n* = 636)	*p*-Value
GA (weeks) ^a^	27 (26–29)	29 (27–31)	<0.001
Birth weight (grams) ^a^	885 (770–1150)	1190 (880–1470)	0.003
Birth weight compared to GA, *n* (%)			0.57
Appropriate for GA	25 (80.6)	537 (84.4)	
Small for GA	5 (16.1)	82 (12.9)	
Large for GA	1 (3.2)	17 (2.7)	
Multifetal gestation, *n* (%)	12 (38.7)	177 (27.8)	0.27
Gestational diabetes mellitus, *n* (%)	1 (3.2)	46 (7.2)	0.71
Pregnancy-induced hypertension, *n* (%)	4 (12.9)	138 (21.7)	0.33
Antenatal steroid use, *n* (%)	23 (74.2)	483 (75.9)	0.97
Preterm premature rupture of membranes, *n* (%)	7 (22.6)	75 (11.9)	0.09
Chorioamnionitis, *n* (%)	2 (6.5)	12 (1.9)	0.14
Cesarean section, *n* (%)	21 (67.7)	475 (74.7)	0.51
Male sex, *n* (%)	18 (58.1)	353 (55.5)	0.92
1 min Apgar score ^a^	3 (1–6)	6 (4–8)	<0.001
5 min Apgar score ^a^	6 (3–7)	8 (7–9)	<0.001
Respiratory distress syndrome severity, *n* (%)			0.002
Mild	7 (22.6)	327 (51.4)	
Moderate	9 (29.0)	156 (24.5)	
Severe	15 (48.4)	153 (24.1)	
Surfactant administration, *n* (%)	23 (74.2)	287 (45.1)	0.003

GA, gestational age. ^a^ median (interquartile range).

**Table 2:** Risk factors associated with persistent pulmonary hypertension of the newborn (PPHN): matched case–control data.

Risk Factor	Neonate with PPHN (*n* = 31)	Neonate Without PPHN (*n* = 124)	*p*-Value
GA (weeks) ^a^	27 (26–29)	27 (26–29)	>0.99
Birth weight (grams) ^a^	885 (770–1150)	918 (764–1195)	0.91
Birth weight compared to GA, *n* (%)			0.30
Appropriate for GA	25 (80.6)	107 (86.3)	
Small for GA	5 (16.1)	16 (12.9)	
Large for GA	1 (3.2)	1 (0.8)	
Multifetal gestation, *n* (%)	12 (38.7)	35 (28.2)	0.36
Gestational diabetes mellitus, *n* (%)	1 (3.2)	7 (5.6)	0.99
Pregnancy-induced hypertension, *n* (%)	4 (12.9)	24 (19.4)	0.57
Antenatal steroid use, *n* (%)	23 (74.2)	88 (71.0)	0.94
Preterm premature rupture of membranes, *n* (%)	7 (22.6)	17 (13.7)	0.27
Chorioamnionitis, *n* (%)	2 (6.5)	3 (2.4)	0.26
Cesarean section, *n* (%)	21 (67.7)	82 (66.1)	>0.99
Male sex, *n* (%)	18 (58.1)	73 (58.9)	>0.99
1 min Apgar score ^a^	3 (1–6)	5 (2–7)	0.04
5 min Apgar score ^a^	6 (3–7)	7 (4–9)	0.03
Respiratory distress syndrome severity, *n* (%)			0.43
Mild	7 (22.6)	39 (31.5)	
Moderate	9 (29.0)	40 (32.3)	
Severe	15 (48.4)	45 (36.3)	
Surfactant administration, *n* (%)	23 (74.2)	77 (62.1)	0.29

GA, gestational age. ^a^ median (interquartile range).

**Table 3:** Clinical outcomes associated with persistent pulmonary hypertension of the newborn (PPHN).

Outcome	Neonate with PPHN (*n* = 31)	Neonate Without PPHN (*n* = 124)	*p*-Value
Composite outcome: mortality and major morbidities, *n* (%)	27 (87.1)	55 (44.4)	<0.001
Mortality, *n* (%)	14 (45.2)	25 (20.2)	0.008
Major morbidities, *n* (%)	17 (54.8)	36 (29.0)	0.01
Moderate or severe bronchopulmonary dysplasia, *n* (%)	10 (32.3)	28 (22.6)	0.39
Postnatal steroid use for bronchopulmonary dysplasia, *n* (%)	8 (25.8)	20 (16.1)	0.32
Treated retinopathy of prematurity, *n* (%)	2/15 (13.3)	3/83 (3.6)	0.17
Severe neurological injury, *n* (%)	10/23 (43.5)	14/100 (14.0)	0.003
Ventilator days ^a^	9 (4–31)	5 (2–12)	0.02
Duration of oxygen use (days) ^a^	12 (5–49)	18 (5–46)	0.95
Length of neonatal intensive care unit stay (days) ^a^	28 (7–63)	29 (12–53)	0.99
Length of hospital stay (days) ^a^	39 (7–81)	54 (28–76)	0.33
Daily hospital costs (USD) ^a^	269.1 (192.0–457.2)	172.0 (138.0–217.4)	<0.001

^a^ median (interquartile range).

**Table 4:** Conditional logistic regression analysis of risk factors associated with persistent pulmonary hypertension of the newborn (PPHN).

Risk Factors Associated with PPHN	Adjusted Odds Ratio (95% Confidence Interval)	*p*-Value
BW compared to gestational age		
Appropriate for gestational age	1	
Small for gestational age	1.15 (0.37–3.60)	0.81
Large for gestational age	3.50 (0.18–68.49)	0.41
1 min Apgar score	0.89 (0.70–1.15)	0.38
5 min Apgar score	0.97 (0.77–1.22)	0.78
Surfactant administration	2.96 (0.33–26.21)	0.33

**Table 5:** Multivariable analysis of outcomes associated with persistent pulmonary hypertension of the newborn (PPHN).

Outcomes Associated with PPHN	Adjusted Odds Ratio (95% Confidence Interval)	*p*-Value
Composite outcomes of mortality and major morbidities, *n* (%)	7.51 (2.41–23.40)	<0.001
Mortality, *n* (%)	2.88 (1.06–7.63)	0.04
Major morbidities, *n* (%)	2.99 (1.29–6.95)	0.01
Moderate or severe bronchopulmonary dysplasia, *n* (%)	1.58 (0.64–3.93)	0.32
Treated retinopathy of prematurity, *n* (%)	1.38 (0.11–17.16)	0.80
Severe neurological injury, *n* (%)	4.44 (1.56–12.59)	0.005

## Data Availability

Raw data supporting the conclusions of this study will be provided by the authors upon request. The data are not publicly available due to privacy concerns.
